# Prevalence of common canine digestive problems compared with other health problems in teaching veterinary hospital, Faculty of Veterinary Medicine, Cairo University, Egypt

**DOI:** 10.14202/vetworld.2015.403-411

**Published:** 2015-03-26

**Authors:** Gamal M. H. Rakha, Mounir M. Abdl-Haleem, Haithem A. M. Farghali, Hitham Abdel-Saeed

**Affiliations:** 1 Department of Internal Medicine and Infectious Diseases, Faculty of Veterinary Medicine, Cairo University, Giza, Egypt; 2 Department of Surgery, Faculty of Veterinary Medicine, Cairo University, Giza, Egypt

**Keywords:** canine, causes, digestive problems, Egypt, prevalence

## Abstract

**Aim:**

The present study was conducted to ascertain the prevalence of common digestive problems compared to other health problems among dogs that were admitted to the teaching veterinary hospital, faculty of veterinary medicine, Cairo University, Egypt during 1 year period from January to December 2013. Also, study the effect of age, sex, breeds, and season on the distribution of digestive problems in dogs.

**Materials and Methods:**

A total of 3864 dogs included 1488 apparently healthy (included 816 males and 672 females) and 2376 diseased dogs (included 1542 males and 834 females) were registered for age, sex, breed, and the main complaint from their owners. A complete history and detailed clinical examination of each case were applied to the aids of radiographic, ultrasonographic, and endoscopic examination tools. Fecal examination was applied for each admitted case. Rapid tests for parvovirus and canine distemper virus detection were also performed.

**Results:**

A five digestive problems were commonly recorded including vomiting, diarrhea, concurrent vomiting with diarrhea, anorexia, and constipation with a prevalence (%) of 13.6, 19.1, 10.1, 13.1, and 0.5 respectively while that of dermatological, respiratory, urinary, neurological, cardiovascular, auditory, and ocular problems was 27.9, 10.5, 3.3, 0.84, 0.4, 0.25, and 0.17 (%) respectively. This prevalence was obtained on the basis of the diseased cases. Age and breed had a significant effect on the distribution of digestive problems in dogs (p<0.001). Gender had an effect on the distribution of digestive problems with significant (p≤0.01) while season had a non-significant effect (p>0.05) on the distribution of such problems.

**Conclusion:**

Digestive problems were the highest recorded problems among dogs, and this was the first records for such problems among dogs in Egypt. Age, gender, and breeds had a significant effect on the distribution of the digestive problems in dogs while season had a non-significant effect on the distribution of such problems. The present data enable veterinarians in Egypt to ascertain their needs for diagnostic tools and medication that must be present at any pet clinic.

## Introduction

Dogs are the most successful canids that were kept as pets by many people all over the world irrespective of their social status and most of these dogs were kept as watch, companion animals, and as a guide to handicapped persons. It also had been used in search and as a rescue dogs by police or armed forces [1]. Healthy digestive organs underpin the function of the whole digestive system through digestion and absorption of the nutrients, neutralization of the toxins and elimination of the wastes and unwanted products. The main problem for the majority of gastrointestinal diseases in dogs was that of clinical signs which may include vomiting, diarrhea and weight loss which shared by many conditions that have either primary or secondary effect on the gastrointestinal tract [2]. For these facts, gastrointestinal disorders in dogs were considered as one of the most common and important cause of presentation to clinicians [3,4]. Vomiting and diarrhea were considered as a ways of discharging offending materials and toxins from the digestive system [5]. Although the higher occurrence of digestive problems among dogs in Egypt, limitation of the data about the prevalence of such problems was present, and the needs for diagnostic tools and emergency medications in clinics were still unclear.

The main object of this study was to determine the prevalence of the most common digestive problems compared with other health problems in dogs that were admitted to the teaching veterinary hospital, department of internal medicine and infectious diseases, faculty of veterinary medicine, Cairo University from January to December 2013. Also, determination of the main causes and the effect of age, sex, breeds, and season on the distribution of digestive problems in dogs.

## Materials and Methods

### Ethical approval

The research procedures were approved by the department of internal medicine and infectious diseases, faculty of veterinary medicine, Cairo University, Egypt.

### Study area

The dogs used for this study were those admitted to the teaching veterinary hospital, faculty of veterinary medicine, Cairo University, Giza, Egypt during the 1 year study period from January to December 2013.

### Animals in the study

A total of 3864 dogs (included 2358 males and 1506 females) which belonging to different ages and breeds were included in this study. This number included 2376 diseased dogs (included 1542 males and 834 females), and 1488 apparently healthy dogs (included 816 males and 672 females) which admitted either for vaccination or for general health checkup.

### Clinical examination

All the cases were firstly registered in the registration book for date, age, sex, breed, and complaint of their owners. Detailed clinical examination of each patient was carried out including complete medical, vaccination, dietary, and environmental history. Visual inspection was done, and pulse, respiration rates, and rectal temperature were carefully taken. Also, examination of the different organs and body systems was performed [6].

### Other diagnostic tools

Radiographic examination was applied according to Burk and Feeney [7] using X-ray machine (Fisher imaging^®^, with X-ray tube EMERALD-125, A-045211, Chicago, U.S.A.). Ultrasonography was applied according to Kealy and McAllister [8] using ultrasonographic device (Pie medical^®^ scanner, Maastricht, Netherlandswith macro and micro convex sector transducers 3.5-5 and 5-7.5 MHz). Gastrointestinal endoscopy was applied according to Tams and Rawlings [9] using flexible endoscope (Fujinon^®^ BRO-YP2 Japan) with an endoscopic camera (Lemke^®^ MC 204) for both esophagogastroduodenoscopy and colonoscopy.

### Fecal analysis

Fecal sample of each case was collected and examined immediately during the examination. Gross appearance, direct smear, and floatation technique were applied for the presence of adult worms, parasitic eggs and protozoal oocyst [10]. Fecal swabs from diarrheic dogs suffered from canine parvovirus infection, and corneal, nasal swabs from dogs with canine distemper virus infection were subjected to rapid antigen test kits. The presence of two color bands (test and control) within the result window indicated a positive result.

### Statistical analysis

Data that were collected about age, sex, breed, and season were recorded and to Microsoft Excel 2010^®^ spreadsheet, stored separately and exported to analytical software using Chi-square test. Values of p≤0.05 were considered as statistically significant.

## Results

**Figure-1:**
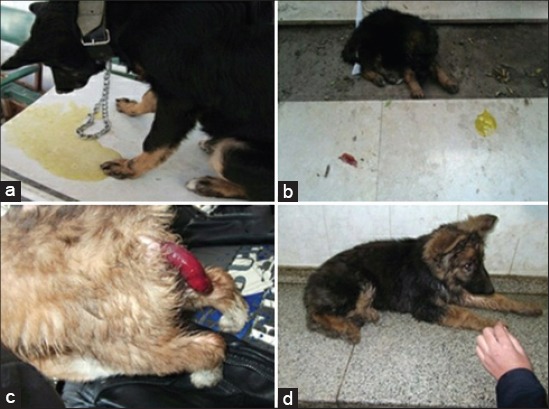
The clinical picture of different digestive problems. (a) Vomiting in 5 month old German Shepherd. (b) German Shepherd puppy suffered from concurrent vomiting with diarrhea as a result of Parvoviral infection. (c) Intestinal intussusception in 3 months old Griffon puppy. (d) 4 months old German Shepherd puppy suffered from generalized weakness, anorexia and dehydration as a result of diarrhea.

**Figure-2:**
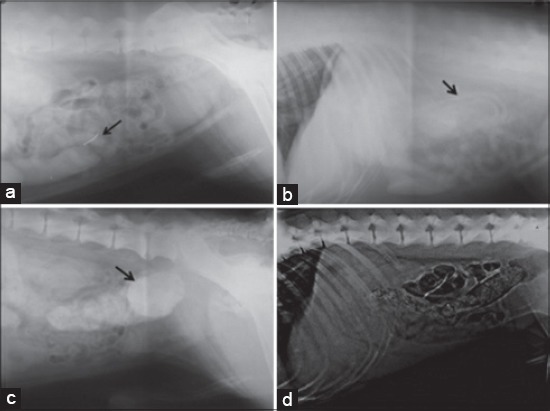
Left lateral radiographic views in some digestive problems among dogs. (a) Sharp foreign body inside the intestine of 8 months old German Shepherd. (b) Thickened intestinal loop due to enteritis in 1 year old Great Dane. (c) Radiopaque stool mass in the colon of 10 months old German Shepherd suffered from severe constipation. (d) Radiopaque pinpoint areas with gases formation inside the colon of 7 months old Rottweiler with signs of constipation and history of ingestion of chicken bones.

**Figure-3:**
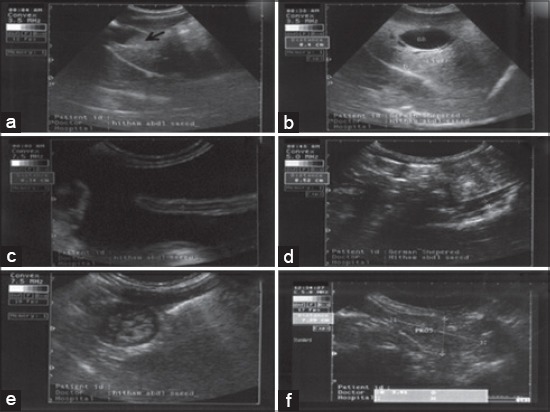
The ultrasonographic findings in dogs with different digestive problems. (a) Biliary sludge (arrow head) in 3 years old Pit Bull suffered from chronic vomiting. (b) Cholecystitis with edema between the two layers of gall bladder wall (0.4 cm) in 2 years old German Shepherd. (c) Free abdominal fluid (ascites) with presence of floating intestinal loop within fluid in 9 month old Pit Bull suffering from concurrent vomiting with diarrhea. (d) Thickened intestinal wall layers (0.52 cm) in 6 months old German Shepherd with diarrhea. (e) Characteristic view (Bull eye) of the intestinal intussusception in 3 months old Griffon suffered from severe obstipation. (f) 3 years old Rottweiler with severe constipation as a result of benign prostatic hyperplasia (measured 7.28 × 3.91 cm).

**Figure-4:**
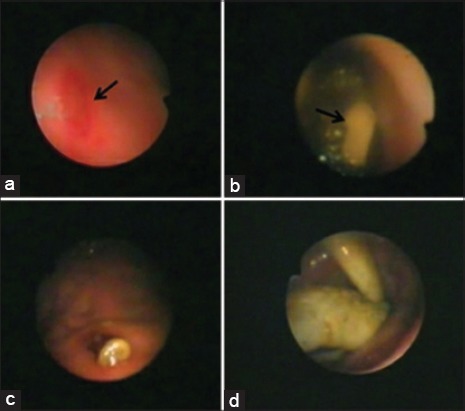
Showed the endoscopic findings of gastrointestinal problems in dogs. (a) Gastric ulcer (arrow head) showed a circumscribed area devoid of mucosa with congestion in 5 years old German Shepherd subjected to prolonged treatment with NSAIDs. (b) *Dipylidium caninum* adult worm within colon (arrow head) in 4-month-old Rottweiler suffered from flea’s infestation and diarrhea. (c) An embedded foreign body in the gastric mucosa of 1-year-old Pit Bull. (d) Colonoscopy showed chicken legs and bones within the colon of 6 months old Golden Retriever suffered from severe constipation and intermittent vomiting.

**Figure-5:**
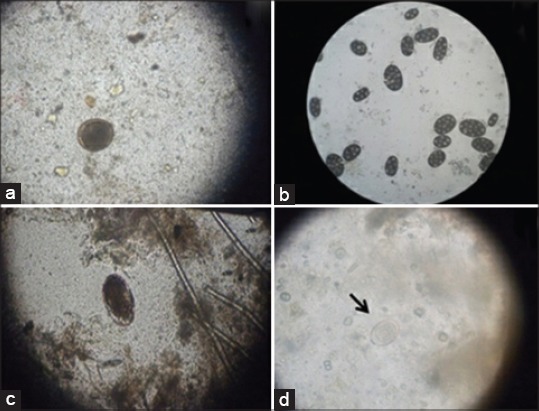
The different diagnosed parasitic eggs and oocysts in the stool of dogs suffered from digestive problems. (a) *Toxocara canis* egg (×100). (b) The egg nest of *Dipylidium caninum* (×100). (c) *Ancylostoma* spp. egg (×100). (d) *Isospora* spp. oocysts (×400).

In the present study, the total number of examined animals was 3864. Out of this number, 2376 animals were diseased, and 1488 animals were apparently healthy representing 61.4% and 38.5% respectively. Diseased animals were divided into cases suffering from digestive problems (1344) and other health problems (1032) representing 56.5% and 43.4% out of diseased animals and 34.8% and 26.7% out of total examined animals respectively. The apparently healthy animals were 1488 which included 954 vaccinated and 534 non-vaccinated dogs representing 64.1% and 35.8% respectively (Table-1). The study showed that there was a five most common occurred digestive problems in dogs included vomiting, diarrhea, concurrent vomiting with diarrhea, anorexia, and constipation in 324, 456, 240, 312, and 12 cases respectively with diseased cases based prevalence of (13.6%), (19.1%), (10.1%), (13.1%), and (0.5%) respectively (Figure-1). Other health problems were included dermatological, respiratory, urinary, neurological, cardiovascular, auditory, and ocular problems and recorded in 662, 250, 80, 20, 10, 6, and 4 cases respectively with diseased number based prevalence of (27.9%), (10.5%), (3.3%), (0.84%), (0.4%), (0.25%), and (0.17%) respectively (Table-2). Regarding the type of feed, historical information revealed that owners offered both commercial dry feed (high or low-quality grade) and homemade diet that were offered all the day. In the present study, 260 cases showed food indiscretion included 185 cases were fed on homemade diets and 75 cases were fed on low grade commercial dry food (Table-3). The major cause of vomiting in the present study was the ingestion of foreign bodies which included chicken bones and fish hooks and this condition was noted in 112 cases and the recorded prevalence was 4.7% from the total diseased cases. Toward diarrhea and concurrent vomiting with diarrhea, the main cause was Parvoviral infection that recorded in 97 and 75 cases respectively with diseased cases based prevalence of 4% and 3.15% respectively. Food indiscretion was the most common cause for anorexia that recorded in 144 cases in the present study (6.1% from diseased cases). In terms of constipation, the most recorded cause was ingestion of foreign bodies and anal sacculitis in 4 cases for each condition with diseased cases based prevalence of 0.16% for each cause (Table-3). The present study showed a significant effect (p<0.001) of age on the occurrence of digestive problems as the puppies up to 6 months of age were more prone to diarrhea than other ages while dogs ranged from 6 months up to 3 years were more prone to anorexia. Older dogs showed an increase in the occurrence of vomiting than other digestive problems (Table-4). Gender had a significant effect (p≤0.01) on the occurrence of digestive problems as males were found to be more affected with diarrhea, vomiting, and concurrent vomiting with diarrhea while females were more prone to anorexia and constipation (Table-5). In terms of breed, there was a significant effect (p<0.001) on the occurrence of digestive problems as vomiting was of higher prevalence in giant and small breeds (Mastiff, Saint Bernard, Great Dane, and Caucasian) and (Griffon, Bug, and Pekingese) respectively. In large breed dogs (German Shepherd, Rottweiler, and Golden Retriever), diarrhea was the most prevalent problem while in medium breed dogs (Pit Bull, Labrador Retriever, and Boxer), anorexia was the most prevalent (Table-6). Season had a non-significant effect (p>0.05) on the distribution of digestive problems among dogs (Table-7). Regarding the diagnostic tools required for definitive diagnosis in the present study, radiographic examination was carried out on 209 cases included 169 cases with foreign bodies, 30 cases with chronic constipation, and 10 cases suffered from renal stones (Figure-2). Ultrasonographic examination was carried out on 236 cases included enteritis, liver diseases, IBD, biliary sludge, renal diseases, pancreatitis, benign prostatic hyperplasia, and intestinal intussusception in 150, 40, 19, 17, 3, 3, 2, and 2 cases respectively (Figure-3). Gastrointestinal endoscopy was performed on 64 cases included 34 cases with foreign bodies, 20 cases with intestinal parasites, and 10 cases with IBD (Figure-4). Rapid tests for detection of parvovirus and canine distemper virus were performed on 412 cases. Out of them, 232 cases showed a positive result that included 172 cases for parvovirus infection and 60 cases for canine distemper viral infection. Fecal analysis showed some parasitic infections included *Toxocara canis, Dipyledium caninum, Ancylostoma spp., and Isospora spp*. (Figure-5).

**Table-1:** The prevalence of digestive problems versus other problems through 1 year period from January to December 2013.

Group	Sub-group	No. of dogs	Prevalence from (%)		

			Diseased group	Apparently Healthy group	Total number
Diseased	Digestive problems	1344	56.5	-	34.8
	Other health problems	1032	43.4	-	26.7
Total	-	2376	-	-	61.4
Apparently Healthy	Vaccinated dogs	954	-	64.1	24.7
	Non-vaccinated dogs	534	-	35.8	13.8
Total	-	1488	-	-	38.5
Total	-	3864	100	100	100

**Table-2:** Common digestive and other health problems in dogs admitted through 1 year period from January to December 2013.

Problem	No. of cases	Prevalence (%) toward			

		Digestive group	Other problems group	Diseased cases	Total no. (3864)
Digestive problems					
Vomiting	324	24.1	-	13.6	8.3
Diarrhea	456	33.9	-	19.1	11.8
Vomiting and Diarrhea	240	17.8	-	10.1	6.2
Anorexia	312	23.2	-	13.1	8
Constipation	12	0.89	-	0.5	0.3
Total	1344				
Other problems					
Dermatological	662	-	64.1	27.9	17.1
Respiratory	250	-	24.2	10.5	6.4
Urinary	80	-	7.7	3.3	2.1
Neurological	20	-	1.9	0.84	0.52
Cardiovascular	10	-	0.96	0.4	0.25
Ear problems	6	-	0.58	0.25	0.15
Ocular	4	-	0.39	0.17	0.1
Total	1032	-	-	-	-
Diseased cases	2376	-	-	-	-

**Table-3:** The prevalence of the common causes of digestive problems in dogs.

Problem	Causes	No.	Prevalence (%) on the basis of			

			Problem cases	Digestive diseased cases	Diseased cases	Total number
Vomiting	Foreign body	112	34.5	8.3	4.7	2.8
	Food indiscretion	60	18.5	4.4	2.5	1.5
	Drugs (NSAIDs)	40	12.3	2.9	1.68	1.03
	Pneumonia	35	10.8	2.6	1.47	0.9
	Liver diseases	30	9.2	2.2	1.26	0.77
	Poisoning	15	4.6	1.1	0.63	0.38
	Biliary sludge	10	3	0.74	0.42	0.25
	Motion sickness	10	3	0.74	0.42	0.25
	IBD	9	2.7	0.66	0.37	0.23
	Renal diseases	3	0.9	0.22	0.12	0.07
Diarrhea	Parvoviral infection	97	21.3	7.2	4	2.51
	*Toxocara* spp.	95	20.8	7.1	3.9	2.4
	*Dipylidium caninum*	80	17.5	5.9	3.3	2
	Food indiscretion	46	10.1	3.4	1.93	1.19
	*Taenia* spp.	41	8.9	3	1.72	1.06
	*Ancylostoma* spp.	35	7.6	2.6	1.47	0.9
	*Isospora* spp.	30	6.5	2.2	1.26	0.77
	Corona viral infection	22	4.8	1.6	0.92	0.56
	IBD	10	2.1	0.74	0.42	0.25
Concurrent vomiting with diarrhea	Parvoviral infection	75	31.3	5.5	3.15	1.94
	Canine distemper	60	25	4.4	2.52	1.55
	Foreign body	53	22	3.9	2.23	1.37
	*Toxocara* spp.	22	9.1	1.6	0.92	0.56
	Food indiscretion	10	4.1	0.74	0.42	0.25
	Liver disease	10	4.1	0.74	0.42	0.25
	Biliary sludge	7	2.9	0.52	0.29	0.18
	Pancreatitis	3	1.25	0.2	0.12	0.07
Anorexia	Food indiscretion	144	46.1	10.7	6.1	3.72
	Oral lesions	120	38.4	8.9	5.1	3.11
	During estrus cycle (Females)	28	8.9	2.1	1.17	0.72
	Pharyngitis	20	6.4	1.4	0.84	0.51
Constipation	Anal sacculitis	4	33.3	0.29	0.16	0.10
	Foreign body	4	33.3	0.29	0.16	0.10
	Benign prostatic hyperplasia	2	16.6	0.14	0.08	0.05
	Intestinal intussusception	2	16.6	0.14	0.08	0.05

**Table-4:** Age-wise prevalence of most common digestive problems in dogs.

Problem	Age (%)						p-value	Chi-square

	1-3 months	3-6 months	6-12 months	12-36 months	>36 months	Total		
Vomiting	192 (27.9)	12 (7.3)	24 (11.6)	60 (28.9)	36 (45)	324	0.00001***	263.31
Diarrhea	264 (38.5)	96 (58.5)	48 (23.2)	24 (11.6)	24 (30)	456		
Vomiting with diarrhea	132 (19.2)	24 (14.6)	24 (11.6)	48 (23.2)	12 (15)	240		
Anorexia	96 (13.9)	30 (18.3)	108 (52.2)	72 (34.8)	6 (7.5)	312		
Constipation	2 (0.29)	2 (1.2)	3 (1.5)	3 (1.5)	2 (2.5)	12		
Total	686	164	207	207	80	1344		

*** p<0.001 (significant)

**Table-5:** Sex-wise prevalence of most common digestive problems in dogs.

Problem	Sex				

	Males (%)	Females (%)	Total	p-value	Chi-square
Vomiting	228 (26.2)	96 (20.3)	324	0.012**	12.65
Diarrhea	300 (34.5)	156 (32.9)	456		
Vomiting with diarrhea	156 (17.9)	84 (17.7)	240		
Anorexia	180 (20.7)	132 (27.8)	312		
Constipation	6 (0.69)	6 (1.3)	12		
Total	870	474	1344		

** p≤0.01 (significant)

**Table-6:** Breed-wise prevalence of most common digestive problems in dogs.

Breeds	Problems						p-value	Chi-square

	Vomiting	Diarrhea	Vomiting and Diarrhea	Anorexia	Constipation	Total		
Giant breed dogs							0.00001***	122.74
Mastiff	15	12	3	13	-	43		
Saint Bernard	3	5	2	5	-	15		
Great Dane	17	12	18	9	1	57		
Caucasian	2	4	5	5	-	16		
Total (%)	37 (28.2)	33 (25.2)	28 (21.4)	32 (24.4)	1 (0.76)	131 (100)		
Large breed dogs								
German Shepherd	96	276	132	96	5	605		
Rottweiler	30	36	24	20	2	104		
Golden Retriever	24	10	20	12	1	67		
Black Coat	12	20	10	40	1	83		
Total (%)	162 (18.6)	342 (39.5)	186 (21.5)	168 (19.4)	9 (1)	867 (100)		
Medium breed dogs								
Pit Bull	48	30	10	48	1	137		
Boxer	20	20	5	24	-	69		
Labrador Retriever	12	3	6	24	-	45		
Doberman	6	-	-	-	-	6		
Dalmatian	-	5	-	-	-	5		
Mixed Breeds	-	-	-	4	-	1		
Total (%)	86 (32.3)	58 (21.8)	21 (7.9)	100 (37.6)	1 (0.38)	266 (100)		
Small breed dogs								
Griffon	33	21	5	12	1	72		
Bug	5	1	-	-	-	6		
Pekingese	1	1	-	-	-	2		
Total (%)	39 (48.8)	23 (28.8)	5 (6.3)	12 (15)	1 (1.3)	80 (100)		
Total	324	456	240	312	12	1344		

*** p<0.001 (significant)

**Table-7:** Season-wise prevalence of most common digestive problems in dogs.

Problems	Season					p-value	Chi-square

	Winter (%)	Spring (%)	Summer (%)	Autumn (%)	Total		
Vomiting	64 (24.8)	70 (20.5%)	120 (27.2)	70 (23.1)	324	0.123	17.74
Diarrhea	81 (31.4)	125 (36.6)	135 (30.5)	115 (37.9)	456		
Vomiting with diarrhea	53 (20.5)	58 (17)	73 (16.5)	56 (18.5)	240		
Anorexia	55 (21.3)	86 (25.2)	112 (25.3)	59 (19.5)	312		
Constipation	5 (1.9)	2 (0.58)	2 (0.45)	3 (0.99)	12		
Total	258	341	442	303	1344		

p>0.05 (Non-significant)

## Discussion

The present study had given an overall idea about the prevalence of the most common occurred digestive problems compared with other problems among dogs which presented for the teaching animal hospital, Faculty of Veterinary Medicine, Cairo University during 1 year period from January to December 2013. The present work showed that digestive problems had a prevalence of 56.5% from the total diseased dogs. Similar findings were reported in a study in Turkey, and the recorded prevalence was 52% [11]. Also, digestive problems had the first order during first observation between 1995 and 1997 in Kosice, Slovakia [12]. Other studies were disagreed with the finding in the present study [13-18]. This difference can be explained as there was a magnificent difference in geographical distribution and the period of the studies. Also, bad feeding management and the endemic nature of most infectious diseases in Egypt such as parvovirus, canine distemper virus infection, and internal parasites infections shared in the elevation of such prevalence. The recorded prevalence of vomiting in the present study was 13.6% from the diseased cases, and this finding was disagreed with Atsbaha *et al*. [18] who recorded a prevalence of 4.6% from diseased cases in Mekelle City, Ethiopia. This may be due to the differences in feeding management, type of feed (when contained bones), and owner’s awareness toward the grade of feed stuff that introduced to their dogs [19]. Also, in some cases, food was subjected to environmental contamination if introduced one time per day making it more prone to be spoiled and decomposed. Another cause was the abuse of anti-inflammatory drugs without any medical recommendation or adjustment of its doses was shared in increase the chance for gastritis and vomiting [20]. The main recorded cause of vomiting in the present work was the ingestion of foreign bodies (Table-3) and this finding was in accordance with Tams and Seim [21]. In agreement with the findings in the present study, diarrhea was the most often registered problem [22]. Several studies were recorded a similar prevalence in the present study [1,18,23]. The recorded prevalence of concurrent vomiting with diarrhea in the present study was 10.1% from the diseased cases, and this result was agreed with Atsbaha *et al*. [18] who recorded a prevalence of 9.1% in a study in Mekelle City, Ethiopia. This could prove that there was a positive association between the occurrence of both diarrhea and vomiting in the same dog while the episodes of both problems didn’t occur at the same time [4]. The most common cause of diarrhea, and concurrent vomiting with diarrhea was the Parvoviral infection, and this finding was agreed with Nappert *et al*. [24]. In terms of anorexia, the recorded prevalence was disagreed with that of Parvez *et al*. [25] and Chaudhari and Atsanda [26]. These differences can be explained as anorexia was the most common presented complaint for many diseases with wide variety of etiology and pathogenesis [27,28]. Constipation was of the lowest prevalence through digestive problems in the present work. This finding explained as constipation wasn’t observed in dogs by many owners [29]. Another cause was that owners tend to treat this condition by traditional methods without resorting to veterinarian. Dermatological related cases were the second major health problems during the study period with recorded prevalence of 27.9% from total diseased cases. This result was in accordance with Jaffri and Rabbani [1] who recorded a prevalence of 22% for such condition in Lahore area, Pakistan while Atsbaha *et al*. [18] and Tarafder and Samad [23] were in disagreement with the study finding and recorded a prevalence of 38.5% and 37.12% respectively. This was attributed to the differences in the geographical distribution and the environmental temperature that controlling most populations of external parasites. The recorded prevalence of respiratory problems was close to that of Atsbaha *et al*. [18] and Yelmaz *et al*. [11] who recorded a prevalence of 13.8% and 8% respectively from diseased cases in Ethiopia and Turkey respectively. Toward urinary problems, Jaffri and Rabbani [1] and Tarafder and Samad [23] recorded a prevalence of 1.27% and 5.15% from diseased cases respectively and these findings were close to that in the present work 3.3%. Another study was disagreed with these findings and recorded this problem in 7.8% of diseases cases [11]. The remaining problems showed a lower prevalence as these conditions were slightly admitted to the clinic. The present study showed a significant effect (p<0.001) of age on the occurrence of digestive problems in dogs. Similar studies demonstrated the same effect [22]. Some workers showed that diarrhea was of higher prevalence in puppies, and it was declined with increasing age [30,31]. This can be explained as the puppies were immunologically inactive and by 12 weeks of age, the majority lost their maternal immunity [32]. Another cause was that stress of weaning, rehoming, and transportation can lead to increase the susceptibility of such problem [33]. Anorexia was a prevalent problem in dogs ranged from 6 to 36 months of age in the present study. This result was attributed to the association between anorexia and many diseases with wide variation of etiology and pathogenesis [27,28]. In older ages (more than 36 months), vomiting was found to be the most prevalent problem in the present study. Most of old dogs were more susceptible to chronic diseases that shared by many clinical signs included vomiting [34]. Other studies reported no association between the age and frequency of gastrointestinal disorders [35]. In the present study a significant effect (p≤0.01) of gender on the occurrence of the digestive problems was found and this finding was in agreement with another study as males were rendered at high risk for developing gastrointestinal disorders [22] especially for diarrhea [35,36]. This result explicated as males had increased sniffing and roaming behavior than females [37,38]. Also, males tend to inspect the head and anogenital areas of other dogs than females [39]. The present study showed that anorexia was more prevalent in females than males. Some workers also detected this result as the pregnancy had some complications that may lead to anorexia [40]. Another explanation was that female sex hormones were found to increase the risk of constipation in females [41]. Some workers were disagreed with these findings and concluded no effect [30,42]. In the present work, breed had a significant effect (p<0.001) on the distribution of digestive problems in dogs. Similar study showed that the incidence of vomiting and diarrhea was significantly affected by breed [22,35]. German Shepherd had a higher prevalence of the gastrointestinal problems than other breeds [23] as there were either differences in genetic susceptibility, feeding regimens, behavior, and the type of feed [22,43]. Regarding season, the present study showed a non-significant effect (p>0.05) of the season on the occurrence of digestive problems in dogs. This finding was in the line with another study showed that diarrhea was commonly detected throughout the year [3]. This can be explained as the symptoms may be seen throughout the year, but the causes for each problem were different. Some studies were disagreed with this finding [22].

## Conclusion

It was the first recorded data about the prevalence of digestive problems and the other health problems for the cases that were admitted to the teaching veterinary hospital, faculty of veterinary medicine, Cairo University, Giza, Egypt during 2013. It was concluded that digestive problems considered as the major health problem among dogs. Age, sex, and breeds had a significant effect on the distribution of digestive problems while season had a non-significant effect on the distribution of such problems in dogs. Integration between diagnostic tools had an effective role in getting a rapid and accurate diagnosis of digestive problems. Medical awareness of the owners should be increased through the clinics toward the feed type (good, bad and toxic feed stuffs), feeding regimens, and quality of feed toward dogs. Great concern should be given to deworming and vaccination status of the dogs with observance the booster vaccination and periodical health checkup. The present data enable the veterinarians in Egypt to ascertain their needs for diagnostic tools and medication that must be present at any pet clinic.

## Authors’ Contributions

GMHR and MMAH designed the entire study. HAMF and HAS carried out all diagnostic procedures included clinical examination, fecal analysis, radiography, ultrasonography, and endoscopy. HAS recorded the prevalence along with analysis of data, finalized the manuscript for communication to the journal. All the authors read the manuscript and approved the final manuscript.
